# A Multiplex PCR Based on Mitochondrial *COI* Sequences for Identification of Members of the *Anopheles barbirostris* Complex (Diptera: Culicidae) in Thailand and Other Countries in the Region

**DOI:** 10.3390/insects11070409

**Published:** 2020-07-02

**Authors:** Parinya Wilai, Rinzin Namgay, Rusdiyah Sudirman Made Ali, Jassada Saingamsook, Atiporn Saeung, Anuluck Junkum, Catherine Walton, Ralph E Harbach, Pradya Somboon

**Affiliations:** 1 Center of Insect Vector Study, Department of Parasitology, Faculty of Medicine, Chiang Mai University, Chiang Mai 50200, Thailand; parinya.wilai@gmail.com (P.W.); jassada_s@cmu.ac.th (J.S.); atiporn.s@cmu.ac.th (A.S.); anuluck.j@cmu.ac.th (A.J.); 2 Vector-Borne Disease Control Programme, Ministry of Health, Gelephu 31101, Bhutan; rinzin69@yahoo.com; 3 Department of Parasitology, Faculty of Medicine, Hasanuddin University, Makassar 90245, Indonesia; dyah.chayank@gmail.com; 4 School of Earth and Environment, Faculty of Science and Engineering, University of Manchester, Manchester M13 9PT, UK; catherine.walton@manchester.ac.uk; 5 Department of Life Sciences, Natural History Museum, Cromwell Road, London SW7 5BD, UK; R.Harbach@nhm.ac.uk

**Keywords:** *Anopheles barbirostris*, *COI*, multiplex PCR, species complex, Thailand

## Abstract

A multiplex-PCR assay based on mitochondrial cytochrome c oxidase subunit I (*COI*) sequences was developed for identification of five members of the Barbirostris Complex which occur in Thailand: *Anopheles barbirostris* s.s., *An. dissidens*, *An. saeungae*, *An. wejchoochotei* and *An. barbirostris* species A3. *Anopheles campestris* was not included in the assay due to the lack of unequivocal sequences. Allele-specific primers were designed for specific nucleotide segments of *COI* sequences of each species. Mismatch method and addition of long GC tail were applied for some primers. The assay provided products of 706 bp for *An. barbirostris* s.s., 238 bp for *An. dissidens*, 611 bp for *An. saeungae*, 502 bp for *An. wejchoochotei* and 365 bp for *An. barbirostris* A3. The assay was tested using 111 wild-caught female mosquitoes from Bhutan, Cambodia, Indonesia (Sulawesi) and Thailand. The results of the multiplex PCR were in complete agreement with *COI* sequencing; however, one of three specimens from Bhutan and all 11 specimens from Indonesia were not amplifiable by the assay due to their distinct *COI* sequences. This, together with the distinct rDNA sequences of these specimens, suggests the presence of at least two additional new species in the Barbirostris Complex.

## 1. Introduction

*Anopheles barbirostris* van der Wulp, a member of the Myzorhynchus Series of the subgenus *Anopheles* Meigen, was formally described in 1884 from a single female collected in eastern Java, Indonesia [1]. Mosquitoes identified morphologically as *An. barbirostris* s.l. are common in the Oriental Region, ranging from Timor, the Indonesian Archipelago, Guam westward across mainland Southeast Asia, southern China and southern Asia [2]. However, genetic and molecular studies revealed that the taxon is a complex of sibling species, the Barbirostris Complex [3], consisting of six formally recognized species: *An. barbirostris* s.s., *An. campestris* Reid, *An. dissidens* Taai and Harbach, *An. saeungae* Taai and Harbach, *An. vanderwulpi* Townson and Harbach and *An. wejchoochotei* Taai and Harbach [4]. The complex includes at least one additional species, i.e., *An. barbirostris* species A3 of Saeung et al. (2008) [5], which is known from Kanchanaburi Province, western Thailand.

The distributions of the members of the complex are not completely known. *Anopheles barbirostris* s.s. is currently known from Indonesia (Java and Kalimantan) and Thailand [6,7]. The role of *An. barbirostris* s.s. as a vector of malarial protozoa is questionable. This species is common in Java, but is not important as a malaria vector due to its strong zoophilic behaviour [8]. In contrast, *An. barbirostris* s.l. in Sulawesi, the Lesser Sunda Island group (Lombok and Flores) and Timor–Leste is more anthropophilic and is an important vector of malarial protozoa and the filarial nematode *Brugia malayi*. Current evidence has revealed that the internal transcribed spacer 2 (ITS2, rDNA) sequence of *An. barbirostris* s.l. from Sulawesi [9] differs from *An. barbirostris* s.s. from Kalimantan [6] by 2.76%, suggesting that it is a distinct species. Laboratory experiments have revealed that *An. barbirostris* s.s. in Thailand is refractory to *Plasmodium falciparum* and *P. vivax* [10]. In Sri Lanka, *An. barbirostris* s.l. is a malaria vector [11], but molecular identification based on *COI* and ITS2 sequences revealed a new molecular type which differs from *An. barbirostris* s.s. by 5.1 and 5.4%, respectively [12]. *Anopheles barbirostris* s.l. is common in India, but the role of this taxon as a malaria vector is unknown [13].

*Anopheles dissidens* is currently known from Thailand, China and Myanmar [4,14]. *Anopheles saeungae* is known from Thailand, Indonesia (Sumatra) and China [4,6,14]. The role of *An. dissidens*, *An. saeungae* and *An. barbirostris* species A3 as natural vectors of malarial parasites is unknown. *Anopheles dissidens* and *An. saeungae* are zoophilic and susceptible to *P. vivax* in the laboratory, with low sporozoite infection rates (<10%) [4,10].

*Anopheles campestris*, first described from specimens collected in Rantau Panjang, Klang, Selangor, Malaysia [15], is an important vector of malarial protozoa and *B. malayi* in peninsular Malaysia [16]. Based on morphological identification, *An. campestris* is also known to occur in Cambodia [17], Thailand [18] and Vietnam [19]. Adults of *An. campestris* differ principally from those of *An. barbirostris* s.s. in having darker scales on the wing veins and more white scales on the abdominal terga; larvae and pupae of the former usually have some abdominal setae with more numerous branches [15]. However, Harrison and Scanlon (1975) [18] reported that these characters were so variable in Thai populations that the two species were difficult to identify with certainty. Baimai et al. (1995) [20] reported that the metaphase karyotype of *An. campestris* from Ayutthaya Province, central Thailand, is distinct from those of *An. barbirostris* s.l. from elsewhere in Thailand and Indonesia (Java). According to Rattanarithikul et al. (2006) [21], *An. campestris* in Thailand appears to be mainly confined to flat rice plains in the central and eastern regions and is absent in hilly areas. This species is often confused with *An. wejchoochotei* (formerly *An. campestris*-like), which is currently only known from Thailand [4], because it is indistinguishable from *An. campestris* in the larval, pupal and adult stages. Unfortunately, no molecular data for *An. campestris* from the type locality is available in GenBank for comparison with *An. campestris* from elsewhere. *Anopheles wejchoochotei* is relatively more anthropophilic and probably a vector of malarial parasites in Sa Kaeo Province in eastern Thailand [22,23]. *Anopheles vanderwulpi* is known from Java and Sumatra of Indonesia and may not be a vector of parasites of human diseases due to its zoophilic behavior [7]. The role of *An. barbirostris* s.l. as a vector of *P. knowlesi*, a primate malarial protozoan, is unknown.

Correct identification of members of the Barbirostris Complex is essential for epidemiology studies and the control of malaria because not all of the sibling species are vectors of agents of human disease. However, members of the complex are difficult to distinguish by morphology due to overlapping morphological characters [4]. Consequently, their distributions and the roles they play in the transmission of disease agents can only be known when specimens are identified by a molecular or cytogenetic method. The latter method, however, is less convenient as it requires laboratory colonies [5,24].

Recently, a double multiplex PCR based on ITS2 sequences was developed for the identification of five members of the Barbirostris Complex in Thailand [25]. However, this method requires two steps of PCR reactions and did not detect *An. barbirostris* species A3, which also occurs in Thailand [5]. Because phylogenetic analyses of the *COI* and ITS2 sequences produced phylogenetic trees with similar topologies [5,6,24,26], in the present study we developed a multiplex PCR method based on *COI* sequences for identification of the species of the Barbirostris Complex that occur in Thailand and other countries in the region, except *An. campestris* due to the lack of available sequences for this nominal taxon in GenBank.

## 2. Materials and Methods 

### 2.1. Mosquitoes

Adult mosquitoes were collected by aspirator in cattle sheds in Bhutan, Cambodia, Sulawesi and Thailand, during 2016–2019 (Table 1). They were killed with chloroform vapour and kept dried in vials with silica gel. Identification was done under a stereomicroscope following Rattanarithikul et al. (2006) [21]. The morphological terminology and abbreviations found in the Anatomical Glossary of the online Mosquito Taxonomic Inventory (http://mosquito-taxonomic-inventory.info/node/11027) are used herein.

### 2.2. DNA Extraction, Amplification and Sequencing of the COI Gene

Genomic DNA was extracted from the legs of individual mosquitoes using the Pure Link™ Genomic DNA Mini Kit (Invitrogen, Carlsbad, CA, USA) according to manufacturer’s instructions. The DNA was eluted with 20 μL (first elusion) followed by 40 μL (second elusion) buffer. Specimens were retained for morphological examination. The mitochondrial *COI* gene was amplified by PCR using the LCO-1490 forward primer (5′-GGTCAACAAATCATAAAGATATTGG-3′) and the HCO-2198 reverse primer (5′-TAAACTTCAGGGTGACCAAAAAATCA-3′) [27]. The 20 μL PCR reaction consisted of 1X PCR buffer (Invitrogen, Carlsbad, CA, USA), 3.0 mM MgCl_2_ (Invitrogen, Carlsbad, CA, USA), 0.2 mM dNTPs (Invitrogen, Carlsbad, CA, USA), 0.4 U Taq DNA polymerase (Invitrogen, Carlsbad, CA, USA), 0.2 μM of each primer and 1 μL (5 ng) of extracted DNA. PCR reaction was conducted by using the Dw-T960 Smart Gradient PCR Thermal Cycler (Drawell, Shanghai, China) and the PCR conditions were as follows: an initial denaturation at 95 °C for 2 min, 40 cycles of denaturation at 95 °C for 30 s, annealing at 45 °C for 30 s and extension at 72 °C for 30 s, and a final extension at 72 °C for 5 min. Amplicons were visualized on 2% agarose gels stained with Ethidium bromide (Invitrogen, Carlsbad, CA, USA). PCR products were purified using the Illustra™ ExoProStar™ 1-Step (GE Healthcare Life Sciences, Buckinghamshire, UK) and sequenced using the BigDye^®^ Terminator v3.1 cycle sequencing kit chemistry (First BASE, Selangor, Malaysia).

### 2.3. Sequencing Alignment and Phylogenetic Analysis

The new *COI* sequences were compared with those of *An. barbirostris* s.l. available in GenBank using the Basic Local Alignment Search Tool (BLAST, available at http://blast.ncbi.nlm.nih.gov/Blast.cgi) under default parameters. GenBank included sequences from Thailand: *An. barbirostris* s.s. (AB373942.1–AB373944.1), *An. dissidens* (LC333239.1, LC333240.1), *An. saeungae* (AB971326.1, AB971327.1), *An. wejchoochotei* (AB971339.1, AB971340.1) and *An. barbirostris* A3 (AB362238.1–AB362240.1). In addition, *COI* sequences listed as *An. campestris* from Chiang Mai (AB436105.1) and Sa Kaeo (AB436124.1) Provinces, Thailand, reported by Thongsahuan et al. (2009) [28], *An. barbirostris* s.l. from mainland India (AY729982.1, HM773367.1, MN166188.1, MN25480.1, MN264217.1) and Andaman and Nicobar Islands (MK184151.1, MK184158.1, MK184180.1, MK184190.1, MK184200.1), Singapore (KF564681.1–KF564683.1) and Vietnam (MH425426.1–MH425429.1, MH425437.1) were also included in the phylogenetic analysis. One specimen of *An. vanderwulpi* from the Natural History Museum, London, was sequenced for *COI* and included in the tree. 

Sequences were aligned using Clustal W version 2.0 under default parameters [29]. Ragged ends were trimmed using MEGA version 10.0.5 [30]. Phylogenetic analyses were conducted using maximum likelihood (ML) with MEGA version 10.0.5 [30]. *Anopheles pullus* Yamada was used as the outgroup, which belongs to the Hyrcanus Group of the Myzorhynchus Series. The best evolutionary model of nucleotide substitution that fits the data was obtained using jModelTest v. 2.1.10 [31]. Robustness of the ML tree was tested with 1000 bootstrapped data sets with bootstrap support values of 70% or more indicated on the tree. This widely used level of bootstrap support [32] was chosen to allow full consideration of how clades inferred in this way correspond to putative species, given that when the latter are closely related they are not expected to have high levels of bootstrap support from a single gene. 

### 2.4. Primer Design and Allele-Specific PCR

Allele-specific primers were designed from published mitochondrial *COI* sequences available in GenBank: *An. barbirostris* s.s. (AB373942–AB373944), *An. dissidens* (AB331574–AB3315780), *An. saeungae* (AB435997, AB436002, AB436007, AB436013–AB436015, AB331570–AB331572)*, An. wejchoochotei* (AB971336–AB971338, and AB331582–AB331588 as *An. campestris*) and *An. barbirostris* species A3 (AB362238–AB362240) (see alignments in Supplementary Figure S1). Sequences were aligned using the Clustal W algorithm [33] implemented in MEGA version 10.0.5 [30]. Primers were designed using the web-based Primer3Plus software [34]. Our initial study revealed difficulties in optimizing conditions for multiplex PCR, in particular high annealing temperatures did not show all diagnostic bands whereas low annealing temperatures showed all diagnostic bands, but there were also nonspecific bands. The mismatch technique [35] was then used to increase specificity and reduce background amplification by positioning the mismatches in some allele-specific primers at the third nucleotide from the 3’ end. To distinguish between the amplification products based on size of the products, the long GC tail was attached to the allele-specific primer [36]. Each allele-specific forward primer and the universal *COI* reverse primer (HCO-2198) were tested with definitively identified specimens of Saeung et al. (2007, 2008) [5,24] and Suwannamit et al. (2009) [26] in order to check the length of the amplified fragments and assess primer specificity. PCR was conducted using 20 μL volumes containing 1X PCR buffer (Invitrogen, Carlsbad, CA, USA), 3.0 mM MgCl_2_ (Invitrogen, Carlsbad, CA, USA), 0.2 mM dNTPs (Invitrogen, Carlsbad, CA, USA), 0.4 U Taq DNA polymerase (Invitrogen, Carlsbad, CA, USA), 0.2 μM of each primer and 1 μL (5 ng) of extracted DNA. The PCR reactions were carried out at 95 °C for 2 min, 40 cycles at 95 °C for 30 s, 45 °C for 30 s and 72 °C for 30 s, and a final extension at 72 °C for 5 min. Amplicons were visualised on 2% agarose gels stained with Ethidium bromide (Invitrogen, Carlsbad, CA, USA).

### 2.5. Multiplex PCR and Validation

The reaction mixtures were prepared from 20 μL volumes that contained 0.2 μM of each primer (six primers). PCR reaction mixture, conditions for amplifications and gel electrophoresis were performed in the same way as the allele-specific PCR method. Results of the novel multiplex-PCR assay were validated by comparing with *COI* sequences of feral specimens of the Barbirostris Complex collected from different locations in Bhutan, Cambodia, Indonesia (Sulawesi) and Thailand (Table 1).

## 3. Results

### 3.1. Phylogenetic Analysis

**Figure 1:**
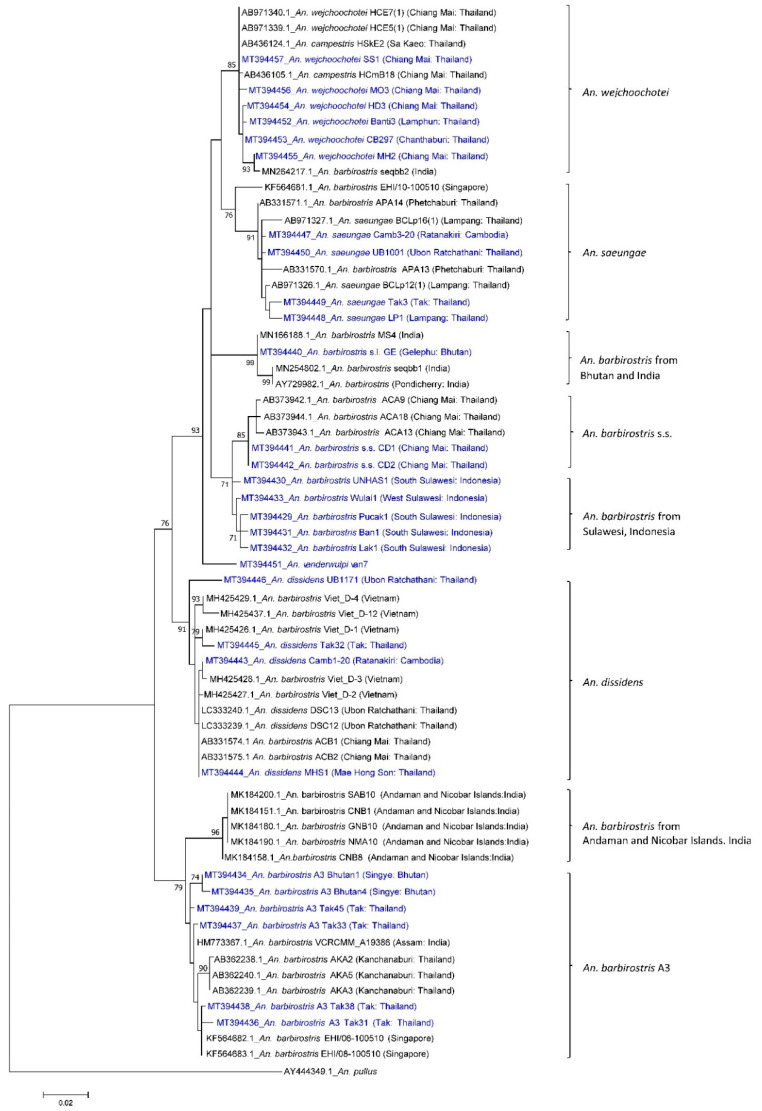
Maximum likelihood tree of *COI* sequences. Representative specimens of *An. barbirostris* s.l. in the current study are highlighted in blue. GenBank sequences included specimens from the Andaman and Nicobar Islands, India, Singapore, Thailand and Vietnam, and *An. pullus* as the outgroup. Bootstrap values of ≥70% are shown.

A total of 111 specimens collected from various locations were sequenced for a fragment of the *COI* gene (Table 1, Supplementary Figure S2). All sequences in this study were deposited in the DDBJ/EMBL/GenBank nucleotide sequence database (MT394429–MT394457, MT450754–MT450836) and Barcode of Life Database (BOLD) (THBAR001-20–THBAR111-20). The best-fit model of nucleotide substitution for ML analysis was GTR+G+I. Twenty-nine representative *COI* sequences from the current study were included in the phylogenetic tree (highlighted as blue in Figure 1). 

Phylogenetic analysis of *COI* sequences (642 bp) revealed that specimens from Thailand collected in the current study were recovered in five clades of the Barbirostris Complex, including *An. barbirostris* s.s., *An. dissidens*, *An. saeungae*, *An. wejchoochotei* and *An. barbirostris* A3, and distinguish from the clades consisting of specimens from Gelephu of Bhutan, Sulawesi, and the Andaman and Nicobar Islands, with supportive bootstrap values (71–99%) (Figure 1). The *An. dissidens* clade also included specimens from Cambodia (MT394443) and GenBank sequences from Vietnam (MH425426.1–MH425429.1, MH425437.1). The sequences of specimens listed as *An. campestris* (AB436105.1, AB436124.1) by Thongsahuan et al. (2009) [28] fell in the *An. wejchoochotei* clade, which also included one GenBank sequence of *An. barbirostris* s.l. from India (MN264217.1). One specimen from Cambodia (MT394447) and one GenBank sequence from Singapore (KF564681.1) fell in the *An. saeungae* clade. The *An. barbirostris* A3 clade includes specimens from Tak Province (MT394436–MT394439), Thailand. Interestingly, two specimens from Singye (MT394434, MT394435), Sarpang of Bhutan fell in this clade, which also included one GenBank sequence from India (HM773367.1) and two GenBank sequences from Singapore (KF564682.1, KF564683.1). Five GenBank sequences from the Andaman and Nicobar Islands (MK184151.1, MK184158.1, MK184180.1, MK184190.1, MK184200.1) formed a distinct clade (ML bootstrap value 96%), which is close to the *An. barbirostris* A3 clade. The sole specimen from Gelephu (MT394440), Sarpang of Bhutan and three GenBank sequences from India (AY729982.1, MN166188.1, MN254802.1) formed a distinct clade (ML bootstrap value 99%). Specimens of *An. barbirostris* s.s. formed a distinct clade (ML bootstrap value 85%) which is closely related to specimens from southern and western Sulawesi (MT394429–MT394433). 

### 3.2. Primer Design and Multiplex PCR

**Figure 2:**
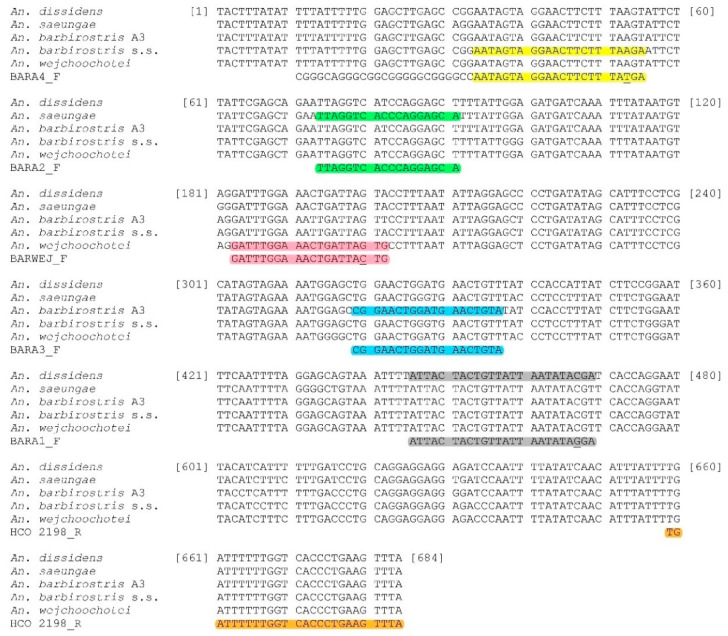
Alignment of *COI* sequences of five species of the Barbirostris Complex. The primer selection sites are highlighted in colors.

Five species-specific reverse primers were designed, based on nucleotide alignment of the complete *COI* region of each species (684 bp) (Figure 2). Primer names, sequences and sizes of the PCR products are shown in Table 2. To increase specificity, mismatches were applied to the third nucleotide from the 3′ end of the allele-specific primers of *An. barbirostris* s.s., *An. dissidens* and *An. wejchoochotei*. Since the allele-specific sites of primers for *An. barbirostris* s.s. and *An. saeungae* were very close, the long GC tail (5′-GCGGGCAGGGCGGCGGGGGCGGGGCC-3′) was attached to the allele-specific primer of *An. barbirostris* s.s. to increase the product size so that the PCR products of both are clearly distinguishable.

**Figure 3:**
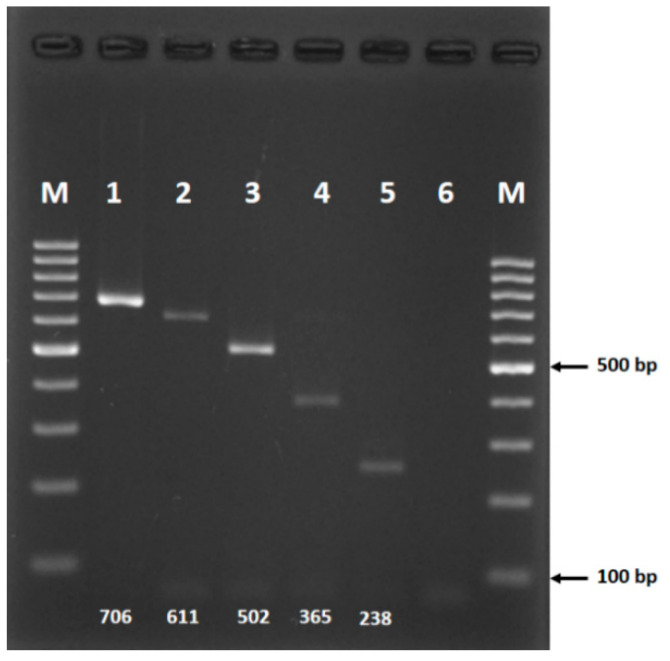
PCR products from the multiplex-PCR assay run on 2% agarose gel. Lanes: 1, *An. barbirostris* s.s.; 2, *An. saeungae*; 3, *An. wejchoochotei*; 4, *An. barbirostris* A3; 5, *An. dissidens*; 6, negative control. The 100-bp molecular weight ladders are loaded in M lanes.

The novel species-specific forward primers that were combined with the universal reverse primer (HCO-2198) successfully simultaneously distinguish all five species of the Barbirostris Complex. This multiplex-PCR assay gave amplified products of 706 bp for *An. barbirostris* s.s., 238 bp for *An. dissidens*, 611 bp for *An. saeungae*, 502 bp for *An. wejchoochotei* and 365 bp for *An. barbirostris* A3 (Table 2, Figure 3). The product bands were clearly visible without nonspecific bands.

### 3.3. Validation

The assay was validated with the 111 sequenced specimens listed in Table 1. The majority of specimens tested were individuals of *An. wejchoochotei* and *An. dissidens*, followed by lesser numbers of *An. barbirostris* species A3 and *An. saeungae*, and only two specimens of *An. barbirostris* s.s. Most specimens were successfully amplified without ambiguous bands, as indicated in Figure 3, the exceptions being one specimen from Gelephu, Bhutan, and 11 specimens from Sulawesi, Indonesia, which have sequences different from those of known members of the Barbirostris Complex (Supplementary Figure S2) and form distinct clades (Figure 1). The results from the multiplex PCR and DNA sequencing for amplifiable *COI* products were in complete agreement. 

## 4. Discussion

Multiplex PCR assays using mtDNA sequences have been used successfully for the identification of members of species complex, e.g., the *An. culicifacies* complex [37]. However, there may be limitations to its use for identification of closely related species due to incomplete lineage sorting and cross-species hybridization events that can result in shared mitochondria between sibling species, e.g., siblings of the *An. lindesayi* complex [38]. The multiplex-PCR assay based on *COI* sequences developed in the present study is relatively simple, fast and cheap compared with DNA sequencing and the multiplex-PCR assay based on ITS2 sequences [25] because it requires only one reaction. The cost of our assay per specimen is about 5 and 10 times cheaper, and 1.2 and 50 times faster, than allele-specific PCR and DNA sequencing, respectively. The designed allele-specific primers and PCR conditions are reliable for identification of specimens of the five species of the Barbirostris Complex that occur in Thailand, including some specimens from Cambodia and Bhutan. The method should facilitate identification of a large number of specimens from Thailand and neighbouring countries, and, thus, should increase knowledge of the distributions of species of the complex and elucidation of the roles they play in the transmission of pathogens. However, there are relatively few sequences for validation of some species, particularly *An. barbirostris* s.s., *An. saeungae* and *An. barbirostris* A3; hence, more specimens from various geographical areas are needed to confirm the reliability of our assay. In addition, the limits of detection depend on the quality of field-caught specimens (such as age and the method of preservation). When freshly captured specimens are used, one or two legs are sufficient to obtain a DNA product and a quantity as low as 1 ng of DNA is amplifiable. In our assay, 5 ng of DNA was generally used with no problem for amplification. However, if there had been no amplified product, the first action to be taken would be to increase the quantity of DNA, up to 10 ng. If there was no product at all, which could be due to poor DNA quality, intraspecific variation or a different species, DNA sequencing would be necessary.

In this study, however, we did not include *An. vanderwulpi*, which occurs in Indonesia, nor did we include *An. campestris* due to the lack of unequivocal *COI* sequences in GenBank. The *COI*, *COII* and ITS2 sequences of *An. wejchoochotei* [4], which were derived from the *An. campestris*-like specimens collected in Sa Kaeo, Chiang Mai and other provinces in Thailand, were often listed as *An. campestris* in GenBank accessions (e.g., Saeung et al., 2007 [24]; Suwannamit et al., 2009 [26]; Thongsahuan et al. 2009 [28] and Paredes-Esquivel et al., 2009 [6]), and this name was subsequently used in publications (e.g., Townson et al., 2013 [7]; Gajapathy et al., 2014 [12]; Sum et al., 2014 [39]). In recent years, attempts to collect and identify *An. campestris* in Thailand based on the metaphase karyotyping reported by Baimai et al. [20], were unsuccessful [5,24,26,28]. The multiplex-PCR assay based on ITS2 sequences developed by Brosseau et al. (2019) [25] included a specific primer designed from a single specimen collected in Chanthaburi Province of eastern Thailand that was identified morphologically. However, its ITS2 sequence has not been deposited in GenBank and whether it corresponds to *An. campestris* from the type locality in Malaysia is not known. Their assay was tested with mosquitoes collected throughout Thailand, but none were amplified by that primer. We also tested four specimens (kindly provided by *T. Chareonviriyaphap*) from the same area in Chanthaburi Province (see Table 1), but they were *An. wejchoochotei*, which appears to be common in Thailand, in agreement with Brosseau et al. (2019) [25]. Based on current evidence, *An. campestris* may be absent or very rare in Thailand. 

Interestingly, the ITS2 sequences of 12 specimens from Selangor, Malaysia included in the phylogenetic analysis of Sum et al. (2014) [39] formed a distinct clade within a larger clade of sequences from sibling species of the Barbirostris Complex collected in Thailand and Indonesia. The larger clade, including the Selangor specimens, comprised five strongly supported clades: three plus the clade of Selangor specimens were labelled as “*An. barbirostris*” and one clade was labelled as *An. campestris*. However, as mentioned above, the clade considered to be *An. campestris* consists of sequences of *An. wejchoochotei* from Thailand, and the clade of Selangor specimens is an unrecognised species. Since Selangor includes the type locality of *An. campestris* [15], it seems quite possible that the specimens of Sum et al. were *An. campestris*, a possibility that needs to be confirmed by further study. 

We also did not test our assay with many specimens of *An. barbirostris* s.s., which appears to be rare in Thailand and mainland Asia. *Anopheles barbirostris* s.s. has not been detected in China [13], peninsular Malaysia [39] and Sri Lanka [12]. We also did not find evidence that this species occurs in Bhutan, Cambodia, India, Singapore and Vietnam. *Anopheles dissidens*, *An. saeungae* and *An. wejchoochotei* are more common in Thailand and other countries in Asia [4,14,19]. Apart from Thailand, China and Myanmar, our study shows that *An. dissidens* also occurs in Cambodia and Vietnam. *Anopheles saeungae* is now known from Singapore in addition to Indonesia (Sumatra), China and Thailand. Besides Thailand, *Anopheles wejchoochotei* is now known from India. *Anopheles barbirostris* species A3 was originally found in Kanchanaburi Province of western Thailand, based on metaphase karyotypes, cross-mating experiments and DNA sequencing [5,26]. This species may have a wider distribution in Asia as it has been detected in Tak Province located immediately north of Kanchanaburi Province, and also Bhutan, India and Singapore. 

The results of the current study suggest the presence of additional new species in the Barbirostris Complex. The first is the specimen from Gelephu, Bhutan, which has its *COI* sequence distinct from those of known members of the complex. Also, its ITS2 sequence differs (>10%) from those of other members of the complex (P. Somboon, unpublished). This putative species is also detected in India. The second is the specimens from Sulawesi, which are closely related to *An. barbirostris* s.s., having *COI* sequences that are distinct from those of other members of the complex. Moreover, their ITS2 sequences are different from *An. barbirostris* s.s. (Kimura 2 parameter genetic distances 2.4–3.2%, unpublished data), agreeing with the different ITS2 sequence (MN203102.1) reported by Davidson et al. (2019) [9]. The distinctiveness of both CO1 and ITS2 has previously indicated that *An. barbirostris* s.l. from Sri Lanka is a distinct species [12]. *An. barbirostris* s.l. from the Andaman and Nicobar Islands, shown here to be closely related to but genetically distinct from *An. barbirostris* A3, could be a further species, but further studies are required to distinguish this from the possibility that it is a geographically distinct population of *An. barbirostris* A3.

## 5. Conclusions

The multiplex PCR based on *COI* sequences is a reliable assay for identification of five of the six species of the Barbirostris Complex, i.e., *An. barbirostris* s.s., *An. dissidens*, *An. saeungae*, *An. wejchoochotei* and *An. barbirostris* species A3. In places like Thailand where *An. campestris* may be rare or absent, our multiplex-PCR assay is unlikely to be compromised. Further, as the two putative new species in Bhutan and Sulawesi based on distinct *COI* sequences were not amplified by our allele-specific primers, their presence would not compromise the validity of the test for identification of the five species for which it is designed. When applying the test to specimens from a new location, it would be advisable to do some initial DNA sequencing to make sure that only these five species are present. In some countries where the five species do not occur, modifications of the assay should be performed to detect those species that are present. However, it is understood that unrecognised species may not produce a PCR product in electrophoresis; hence, further collections, DNA sequencing and phylogenetic analyses are required in these cases.

## Figures and Tables

**Table 1:** Details of collections of *An. barbirostris* s.l. made in Bhutan, Cambodia, Indonesia and Thailand, and comparison of results from multiplex PCR and mitochondrial cytochrome c oxidase subunit I (*COI*) sequencing.

Country	Location	Location Code	Coordinates	Multiplex PCR /*COI* Sequences (No. of Specimens)						
				*An. barbirostris* s.s.	*An. dissidens*	*An. saeungae*	*An. wejchoochotei*	*An. barbirostris* A3	Unknown	Total
Thailand	Mae On, Chiang Mai	MO	18°44′45″ N 99°13′27″ E				7/7			7/7
	Hang Dong, Chiang Mai	HD	18°43′45″ N 98°54′22″ E				3/3			3/3
	San Sai, Chiang Mai	SS	18°59′09″ N 99°05′11″ E				6/6			6/6
	Chiang Dao, Chiang Mai	CD	19°23′27″ N 98°55′51″ E	2/2						2/2
	Mueang Chiang Mai, Chiang Mai	MH	18°45′21″ N 98°56′22″ E				2/2			2/2
	Ko Kha, Lampang	LP	18°07′44″ N 99°20′43″ E			2/2				2/2
	Ban Thi, Lamphun	Banti	18°39′16″ N 99°10′04″ E				8/8			8/8
	Mae Sariang, Mae Hong Son	MS	18°09′24″ N 97°56′15″ E		3/3					3/3
	Tha Song Yang, Tak	Tak	17°27′05″ N 98°10′40″ E		3/3	1/1		9/9		13/13
	Makham, Chanthaburi	CB	12°40′17″ N 102°11′51″ E				4/4			4/4
	Na Chaluai, Ubon Ratchathani	UB	14°32′54″ N 105°14′33″ E		38/38	3/3				41/41
Cambodia	Ratanakiri	Camb	13°51′26″ N 107°06′04″ E		5/5	1/1				6/6
Bhutan	Singye, Sarpang	Bhutan	26°50′44″ N 90°12′26″ E					2/2		2/2
	Gelephu, Sarpang	GE	26°54′47″ N 90°30′07″ E						0/1	0/1
Indonesia	Bantaeng, South Sulawesi	Ban	05°26′58″ S 119°54′15″ E						0/1	0/1
	Makassar, South Sulawesi	Lak	05°07′20″ S 119°28′05″ E						0/5	0/5
		UNHAS	05°07′54″ S 119°29′02″ E						0/2	0/2
	Maros, South Sulawesi	Pucak	05°09′29″ S 119°42′01″ E						0/2	0/2
	Wulai, West Sulawesi	Wulai	01°02′42″ S 119°31′27″ E						0/1	0/1
	Total			2/2	49/49	7/7	30/30	11/11	0/12	99/111

**Table 2:** Primers designed for the multiplex-PCR assay. Long GC tail was added to the allele-specific primer for *An. barbirostris* s.s. Mismatches of nucleotide bases are underlined.

Species	Primer Name	Primer Sequence (5′–3′)	Product Size (bp)
*An. barbirostris* s.s.	BARA4_F	[long GC tail] ^a^ AATAGTAGGAACTTCTTTATGA	706
*An. dissidens*	BARA1_F	ATTACTACTGTTATTAATATAGGA	238
*An. saeungae*	BARA2_F	TTAGGTCACCCAGGAGCA	611
*An. wejchoochotei*	BARWEJ_F	GATTTGGAAACTGATTACTG	502
*An. barbirostris* A3	BARA3_F	CGGAACTGGATGAACTGTA	365
Universal reverse primer	HCO 2198_R	TAAACTTCAGGGTGACCAAAAAATCA	

^a^ Long GC tail sequence: 5′-GCGGGCAGGGCGGCGGGGGCGGGGCC-3′.

## Supplementary Materials

The following are available online at https://www.mdpi.com/2075-4450/11/7/409/s1: Figure S1. Alignments of *COI* sequences of *An. barbirostris* s.l. from GenBank used for designing specific primers for *An. barbirostris* s.s. (yellow), *An. saeungae* (green), *An. wejchoochotei* (pink), *An. barbirostris* A3 (blue) and *An. dissidens* (gray). Dots (·) indicate identity of nucleotides within the alignment; Figure S2. Alignments of the *COI* sequences of the 111 specimens and GenBank sequences included in the study. Dashes (–) indicate alignment gaps and dots (·) indicate identity of nucleotides within the alignment.
