# Supplementary material for: A Multiplex PCR Based on Mitochondrial COI Sequences for Identification of Members of the Anopheles barbirostris Complex (Diptera: Culicidae) in Thailand and Other Countries in the Region
Source: Insects. 2020 Jul 2;11(7):409. doi: 10.3390/insects11070409 (PMC7412068; doi:10.3390/insects11070409)
Supplement: Supplementary file 1 [file insects-11-00409-s001.pdf]

Figure S1. Alignments of *COI* sequences of *An. barbirostris* s.l. from GenBank used for designing specific primers for *An. barbirostris* s.s. (yellow), *An. saeungae* (green), *An. wejchoochotei* (pink), *An. barbirostris* A3 (blue) and *An. dissidens* (gray). Dots (·) indicate identity of nucleotides within the alignment.

	1	1111111112	2222222223	3333333334	4444444445	5555555556
	1234567890	1234567890	1234567890	1234567890	1234567890	1234567890
AB331574.1 *An. barbirostris* ACB1	TACTTTATAT	TTTATTTTTG	GAGCTTGAGC	CGGAATAGTA	GGAACTTCTT	TAAGTATTCT
AB331575.1 *An. barbirostris* ACB2	....	....	....	....	....	....
AB331576.1 *An. barbirostris* ACB3	....	....	....	....	....	....
AB331577.1 *An. barbirostris* AUB2	....	....	....	....	....	....
AB331578.1 *An. barbirostris* AUB6	....	....	....	....	....	....
AB331579.1 *An. barbirostris* AUB10	....	....	....	....	....	....
AB331580.1 *An. barbirostris* AUB11	....	....	....	....	....	....
AB435997.1 *An. barbirostris* ALpA1	....	....	....	A.........	....	....
AB436002.1 *An. barbirostris* AUdA8	....	....	....	A.........	....	....
AB436007.1 *An. barbirostris* AUbB1	....	....	....	A.........	....	....
AB436014.1 *An. barbirostris* ARbA3	....	....	....	A.........	....	....
AB436015.1 *An. barbirostris* ARbB4	....	....	....	A.........	....	....
AB331570.1 *An. barbirostris* APA13	....	....	....	A.........	....	....
AB331571.1 *An. barbirostris* APA14	....	....	....	A.........	....	....
AB331572.1 *An. barbirostris* APA18	....	....	....	A.........	....	....
AB362238.1 *An. barbirostris* AKA2	....	....	....	....	....	....
AB362239.1 *An. barbirostris* AKA3	....	....	....	....	....	....
AB362240.1 *An. barbirostris* AKA5	....	....	....	....	....	....
AB373942.1 *An. barbirostris* ACA9	....	....	....	....	....	....A.....
AB373943.1 *An. barbirostris* ACA13	....	....	....	....	....	....A.....
AB373944.1 *An. barbirostris* ACA18	....	....	....	....	....	....A.....
AB331582.1 *An. campestris* HCB9	....	....	....	....	....	....
AB331583.1 *An. campestris* HCB10	....	....	....	....	....	....
AB331584.1 *An. campestris* HCB13	....	....	....	....	....	....
AB331585.1 *An. campestris* HCE6	....	....	....	....	....	....
AB331586.1 *An. campestris* HCE7	....	....	....	....	....	....
AB331587.1 *An. campestris* HCE8	....	....	....	....	....	....
AB331588.1 *An. campestris* HCE16	....	....	....	....	....	....
AB971336.1 *An. wejchoochotei* HCE2(1)	....	....	....	....	....	....
AB971337.1 *An. wejchoochotei* HCE3(1)	....	....	....	....	....	....
AB971338.1 *An. wejchoochotei* HCE4(1)	....	....	....	....	....	....

				1	1111111111	1111111111
	6666666667	7777777778	8888888889	9999999990	0000000001	1111111112
	1234567890	1234567890	1234567890	1234567890	1234567890	1234567890
AB331574.1 *An. barbirostris* ACB1	TATTCGAGCA	GAATTAGGTC	ATCCAGGAGC	TTTTATTGGA	GATGATCAAA	TTTATAATGT
AB331575.1 *An. barbirostris* ACB2	....	....	....	....	....	....
AB331576.1 *An. barbirostris* ACB3	....	....	....	....	....	....
AB331577.1 *An. barbirostris* AUB2	....	....	....	....	....	....
AB331578.1 *An. barbirostris* AUB6	....	....	....	....	....	....
AB331579.1 *An. barbirostris* AUB10	....	....	....	....	....	....
AB331580.1 *An. barbirostris* AUB11	....	....	....	....	....	....
AB435997.1 *An. barbirostris* ALpA1	.........T	....	.C........	A.........	....	....
AB436002.1 *An. barbirostris* AUdA8	.........T	....	.C........	A.........	....	....
AB436007.1 *An. barbirostris* AUbB1	.........T	....	.C........	A.....G...	....	....
AB436014.1 *An. barbirostris* ARbA3	.........T	....	.C........	A.........	....	....
AB436015.1 *An. barbirostris* ARbB4	.........T	....	.C........	A.........	....	....
AB331570.1 *An. barbirostris* APA13	.........T	....	.C........	A.........	....	....
AB331571.1 *An. barbirostris* APA14	.........T	....	.C........	A.........	....	....
AB331572.1 *An. barbirostris* APA18	.........T	....	.C........	A.........	....	....
AB362238.1 *An. barbirostris* AKA2	....	....	....	....	....	....
AB362239.1 *An. barbirostris* AKA3	....	....	....	....	....	....
AB362240.1 *An. barbirostris* AKA5	....	....	....	....	....	....
AB373942.1 *An. barbirostris* ACA9	.........T	....	....	.........G	....	....
AB373943.1 *An. barbirostris* ACA13	.........T	....	....	.........G	....	....
AB373944.1 *An. barbirostris* ACA18	.........T	....	....	.........G	....	....
AB331582.1 *An. campestris* HCB9	.........T	....	....	....	....	....
AB331583.1 *An. campestris* HCB10	.........T	....	....	....	....	....
AB331584.1 *An. campestris* HCB13	.........T	....	....	....	....	....
AB331585.1 *An. campestris* HCE6	.........T	....	....	....	....	....
AB331586.1 *An. campestris* HCE7	.........T	....	....	....	....	....
AB331587.1 *An. campestris* HCE8	.........T	....	....	....	....	....
AB331588.1 *An. campestris* HCE16	.........T	....	....	....	....	....
AB971336.1 *An. wejchoochotei* HCE2(1)	.........T	....	....	....	....	....
AB971337.1 *An. wejchoochotei* HCE3(1)	.........T	....	....	....	....	....
AB971338.1 *An. wejchoochotei* HCE4(1)	.........T	....	....	....	....	....

	1111111111	1111111111	1111111111	1111111111	1111111111	1111111111
	2222222223	3333333334	4444444445	5555555556	6666666667	7777777778
	1234567890	1234567890	1234567890	1234567890	1234567890	1234567890
AB331574.1 *An. barbirostris* ACB1	AATTGTTACA	GCTCATGCTT	TTATTATAAT	TTTTTTTATA	GTTATACCTA	TTATAATTGG
AB331575.1 *An. barbirostris* ACB2	....	....	....	....	....	....
AB331576.1 *An. barbirostris* ACB3	....	....	....	....	....	....
AB331577.1 *An. barbirostris* AUB2	....	....	....	....	..C.......	....
AB331578.1 *An. barbirostris* AUB6	....	....	....	....	....	....
AB331579.1 *An. barbirostris* AUB10	....	....	....	....	....	....
AB331580.1 *An. barbirostris* AUB11	....	....	....	....	....	....
AB435997.1 *An. barbirostris* ALpA1	....	....	....	...C......	....	....
AB436002.1 *An. barbirostris* AUdA8	....	....	....	...C......	....	....
AB436007.1 *An. barbirostris* AUbB1	....	....	....	...C......	..C.......	......G...
AB436014.1 *An. barbirostris* ARbA3	....	....	....	...C......	....	....
AB436015.1 *An. barbirostris* ARbB4	....	....	....	...C......	....	....
AB331570.1 *An. barbirostris* APA13	....	....	G.........	...C......	....	....
AB331571.1 *An. barbirostris* APA14	....	....	....	...C......	....	....
AB331572.1 *An. barbirostris* APA18	....	....	....	...C......	....	....
AB362238.1 *An. barbirostris* AKA2	....	....	....	C..C......	..A.......	....
AB362239.1 *An. barbirostris* AKA3	....	....	....	C..C......	..A.......	....
AB362240.1 *An. barbirostris* AKA5	....	....	....	C..C......	..A.......	....
AB373942.1 *An. barbirostris* ACA9	....	....	....	...C......	....	....
AB373943.1 *An. barbirostris* ACA13	....	....	....	...C......	....	....
AB373944.1 *An. barbirostris* ACA18	....	....	....	...C......	....	....
AB331582.1 *An. campestris* HCB9	....	....	....	...C......	....	....
AB331583.1 *An. campestris* HCB10	....	....	....	...C......	....	....
AB331584.1 *An. campestris* HCB13	....	....	....	...C......	..C.......	....
AB331585.1 *An. campestris* HCE6	....	....	....	...C......	....	....
AB331586.1 *An. campestris* HCE7	....	....	....	...C......	....	....
AB331587.1 *An. campestris* HCE8	....	....	....	...C......	....	....
AB331588.1 *An. campestris* HCE16	....	....	....	...C......	....	....
AB971336.1 *An. wejchoochotei* HCE2(1)	....	....	....	...C......	....	....
AB971337.1 *An. wejchoochotei* HCE3(1)	....	....	....	...C......	....	....
AB971338.1 *An. wejchoochotei* HCE4(1)	....	....	....	...C......	....	....

	1111111111	1111111112	2222222222	2222222222	2222222222	2222222222
	8888888889	9999999990	0000000001	1111111112	2222222223	3333333334
	1234567890	1234567890	1234567890	1234567890	1234567890	1234567890
AB331574.1 *An. barbirostris* ACB1	AGGATTTGGA	AACTGATTAG	TACCTTTAAT	ATTAGCAGCC	CCTGATATAG	CATTTCCTCG
AB331575.1 *An. barbirostris* ACB2	....	....	....	....	....	....
AB331576.1 *An. barbirostris* ACB3	....	....	....	....	....	....
AB331577.1 *An. barbirostris* AUB2	....	....	....	....	....	....
AB331578.1 *An. barbirostris* AUB6	....	....	....	....	....	....
AB331579.1 *An. barbirostris* AUB10	....	....	....	....	....	....
AB331580.1 *An. barbirostris* AUB11	....	....	....	....	....	....
AB435997.1 *An. barbirostris* ALpA1	G.........	....	....	....	....	....
AB436002.1 *An. barbirostris* AUdA8	G.........	....	....	....	....	....
AB436007.1 *An. barbirostris* AUbB1	G.........	....	....	....	....	....
AB436014.1 *An. barbirostris* ARbA3	G.........	....	....	....	....	....
AB436015.1 *An. barbirostris* ARbB4	G.........	....	....	....	....	....
AB331570.1 *An. barbirostris* APA13	G.........	....	....	....	....	....
AB331571.1 *An. barbirostris* APA14	....	....	....	....	....	....
AB331572.1 *An. barbirostris* APA18	G.........	....	....	....	....	....
AB362238.1 *An. barbirostris* AKA2	....	..T.......	.T........	.........T	....	....
AB362239.1 *An. barbirostris* AKA3	....	..T.......	.T........	.........T	....	....
AB362240.1 *An. barbirostris* AKA5	....	..T.......	.T........	.........T	....	....
AB373942.1 *An. barbirostris* ACA9	....	..T.......	....	.........T	....	....
AB373943.1 *An. barbirostris* ACA13	....	..T.......	....	.........T	....	....
AB373944.1 *An. barbirostris* ACA18	....	..T.......	...T......	.........T	....	....
AB331582.1 *An. campestris* HCB9	....	....	.G........	.........A	....	....
AB331583.1 *An. campestris* HCB10	....	....	.G........	.........T	....	....
AB331584.1 *An. campestris* HCB13	....	....	.G........	.........T	....	....
AB331585.1 *An. campestris* HCE6	....	....	.G........	.........T	....	....
AB331586.1 *An. campestris* HCE7	....	....	.G........	.........T	....	....
AB331587.1 *An. campestris* HCE8	....	....	.G........	.........T	....	....
AB331588.1 *An. campestris* HCE16	....	....	.G........	.........T	....	....
AB971336.1 *An. wejchoochotei* HCE2(1)	....	....	.G........	.........T	....	....
AB971337.1 *An. wejchoochotei* HCE3(1)	....	....	.G........	.........T	....	....
AB971338.1 *An. wejchoochotei* HCE4(1)	....	....	.G........	.........T	....	....

	2222222222	2222222222	2222222222	2222222222	2222222222	2222222223
	4444444445	5555555556	6666666667	7777777778	8888888889	9999999990
	1234567890	1234567890	1234567890	1234567890	1234567890	1234567890
AB331574.1 *An. barbirostris* ACB1	AATAAATAAT	ATAAGATTTT	GAATATTACC	CCCTTCTCTT	ACTTTATTAA	TTTCTAGAAG
AB331575.1 *An. barbirostris* ACB2	....	....	....	....	....	....
AB331576.1 *An. barbirostris* ACB3	....	....	....	....	....	....
AB331577.1 *An. barbirostris* AUB2	....	....	....	....	....	....
AB331578.1 *An. barbirostris* AUB6	....	....	....	T.........	....	....
AB331579.1 *An. barbirostris* AUB10	....	....	....	....	....	....
AB331580.1 *An. barbirostris* AUB11	....	....	....	T.........	....	....
AB435997.1 *An. barbirostris* ALpA1	....	....	....	T.........	....	....
AB436002.1 *An. barbirostris* AUdA8	....	....	....	T.........	....	....
AB436007.1 *An. barbirostris* AUbB1	....	....	....	T.........	...A......	....
AB436014.1 *An. barbirostris* ARbA3	....	....	....	T.........	....	....
AB436015.1 *An. barbirostris* ARbB4	....	....	....	T.........	....	....
AB331570.1 *An. barbirostris* APA13	....	....	....	T.......GC	....	....
AB331571.1 *An. barbirostris* APA14	....	....	....	T.........	....	....
AB331572.1 *An. barbirostris* APA18	....	....	....	T.........	....	....
AB362238.1 *An. barbirostris* AKA2	....	....	....	T.........	....	....
AB362239.1 *An. barbirostris* AKA3	....	....	....	T.........	....	....
AB362240.1 *An. barbirostris* AKA5	....	....	....	T.........	....	....
AB373942.1 *An. barbirostris* ACA9	....	....	.....C....	T.........	....	....
AB373943.1 *An. barbirostris* ACA13	....	....	....	T.........	....	....
AB373944.1 *An. barbirostris* ACA18	....	....	.....C....	T.........	....	....
AB331582.1 *An. campestris* HCB9	....	....	....	T.........	....	....
AB331583.1 *An. campestris* HCB10	....	....	....	T.........	....	....
AB331584.1 *An. campestris* HCB13	....	....	....	T.........	....	....
AB331585.1 *An. campestris* HCE6	....	....	....	T.........	....	....
AB331586.1 *An. campestris* HCE7	....	....	....	T.........	....	....
AB331587.1 *An. campestris* HCE8	....	....	....	T.........	....	....
AB331588.1 *An. campestris* HCE16	....	....	....	T.........	....	....
AB971336.1 *An. wejchoochotei* HCE2(1)	....	....	....	T.........	....	....
AB971337.1 *An. wejchoochotei* HCE3(1)	....	....	....	T.........	....	....
AB971338.1 *An. wejchoochotei* HCE4(1)	....	....	....	T.........	....	....

	3333333333	3333333333	3333333333	3333333333	3333333333	3333333333
	0000000001	1111111112	2222222223	3333333334	4444444445	5555555556
	1234567890	1234567890	1234567890	1234567890	1234567890	1234567890
AB331574.1 *An. barbirostris* ACB1	TATAGTAGAA	AATGGAGCTG	GAACTGGATG	AACTGTTTAT	CCACCATTAT	CTTCCGGAAT
AB331575.1 *An. barbirostris* ACB2	....	....	....	....	....	....
AB331576.1 *An. barbirostris* ACB3	....	....	....	....	....	....
AB331577.1 *An. barbirostris* AUB2	....	....	....	....	....	....
AB331578.1 *An. barbirostris* AUB6	....	....	....	....	....	....
AB331579.1 *An. barbirostris* AUB10	....	....	....	....	....	....
AB331580.1 *An. barbirostris* AUB11	....	....	....	....	....	....
AB435997.1 *An. barbirostris* ALpA1	....	....	.......G..	C	..T..T....	....T.....
AB436002.1 *An. barbirostris* AUdA8	....	....	.......G..	C	..T..T....	....T.....
AB436007.1 *An. barbirostris* AUbB1	....	....	....	C	..T..T....	....
AB436014.1 *An. barbirostris* ARbA3	....	....	.......G..	C	..T..T....	....T.....
AB436015.1 *An. barbirostris* ARbB4	....	....	.......G..	C	..T..T....	....T.....
AB331570.1 *An. barbirostris* APA13	....	....	.......G..	C	..T.......	....
AB331571.1 *An. barbirostris* APA14	....	....	.......G..	C	..T..T....	....T.....
AB331572.1 *An. barbirostris* APA18	....	....	.......G..	C	..T..T....	....T.....
AB362238.1 *An. barbirostris* AKA2	....	........C.	....	......A...	.....T....	....T.....
AB362239.1 *An. barbirostris* AKA3	....	........C.	....	......A...	.....T....	....T.....
AB362240.1 *An. barbirostris* AKA5	....	........C.	....	......A...	.....T....	....T.....
AB373942.1 *An. barbirostris* ACA9	....	........C.	.......G..	....	..T..T....	....T..G..
AB373943.1 *An. barbirostris* ACA13	....	....	.......G..	....	..T..T....	....T..G..
AB373944.1 *An. barbirostris* ACA18	....	....	.......G..	....	..T..T....	....T..G..
AB331582.1 *An. campestris* HCB9	....	.....G....	....	C	..T..T....	....T..G..
AB331583.1 *An. campestris* HCB10	....	.....G....	....	C	..T..T....	....T..G..
AB331584.1 *An. campestris* HCB13	....	.....G....	....	C	..T..T....	....T..G..
AB331585.1 *An. campestris* HCE6	....	.....G....	....	C	..T..T....	....T..G..
AB331586.1 *An. campestris* HCE7	....	.....G....	....	C	..T..T....	....T..G..
AB331587.1 *An. campestris* HCE8	....	.....G....	....	C	..T..T....	....T..G..
AB331588.1 *An. campestris* HCE16	....	.....G....	....	C	..T..T....	....T..G..
AB971336.1 *An. wejchoochotei* HCE2(1)	....	.....G....	....	C	..T..T....	....T..G..
AB971337.1 *An. wejchoochotei* HCE3(1)	....	.....G....	....	C	..T..T....	....T..G..
AB971338.1 *An. wejchoochotei* HCE4(1)	....	.....G....	....	C	..T..T....	....T..G..

```
                                              3333333333 3333333333 3333333333 3333333334 4444444444 4444444444
                                              6666666667 7777777778 8888888889 9999999990 0000000001 1111111112
                                              1234567890 1234567890 1234567890 1234567890 1234567890 1234567890
AB331574.1 An. barbirostris ACB1              TGCACATGCA GGAGCCTCTG TTGATTTAGC TATTTTTTCA TTACATTTAG CAGGAATTTC
AB331575.1 An. barbirostris ACB2              .......... .......... .......... .......... .......... ..........
AB331576.1 An. barbirostris ACB3              .......... .......... .......... .......... .......... ..........
AB331577.1 An. barbirostris AUB2              .......... .......... .......... .......... .......... ..........
AB331578.1 An. barbirostris AUB6              .......... .......... .......... .......... .......... ..........
AB331579.1 An. barbirostris AUB10             .......... .......... .......... .......... .......... ..........
AB331580.1 An. barbirostris AUB11             .......... .......... .......... .......... .......... ..........
AB435997.1 An. barbirostris ALpA1             ...T...... ..G..T.... .......... A......... .......... ..........
AB436002.1 An. barbirostris AUdA8             ...T...... .....T.... .......... A......... .......... ..........
AB436007.1 An. barbirostris AUbB1             ...T...... .....T.... .......... A......... .......... ..........
AB436014.1 An. barbirostris ARbA3             ...T...... .....T.... .......... A......... .......... ..........
AB436015.1 An. barbirostris ARbB4             ...T...... .....T.... .......... A......... .......... ..........
AB331570.1 An. barbirostris APA13             ...T...... .....T.... .......... A......... .......... ..........
AB331571.1 An. barbirostris APA14             ...T...... .....T.... .......... A......... .......... ..........
AB331572.1 An. barbirostris APA18             ...T...... .....T.... .......... A......... .......... ..........
AB362238.1 An. barbirostris AKA2              .......... .....T.... .......G.. .......... .......... ..........
AB362239.1 An. barbirostris AKA3              .......... .....T.... .......... .......... .......... ..........
AB362240.1 An. barbirostris AKA5              .......... .....T.... .......... .......... .......... ..........
AB373942.1 An. barbirostris ACA9              ...T...... .....T.... ........T. A......... .......... ..........
AB373943.1 An. barbirostris ACA13             ...T...... .....T.... ........T. A....A.... .......... ..........
AB373944.1 An. barbirostris ACA18             ...T...... .....T.... ........T. A......... .......... ..........
AB331582.1 An. campestris HCB9                ...T...... .....T.... .......... A......... .......... ..........
AB331583.1 An. campestris HCB10               ...T...... .....T.... .......... A......... .......... ..........
AB331584.1 An. campestris HCB13               ...T...... .....T.... .......... A......... .......... ..........
AB331585.1 An. campestris HCE6                ...T...... .....T.... .......... A......... .......... ..........
AB331586.1 An. campestris HCE7                ...T...... .....T.... .......... A......... .......... ..........
AB331587.1 An. campestris HCE8                ...T...... .....T.... .......... A......... .......... ..........
AB331588.1 An. campestris HCE16               ...T...... .....T.... .......... A......... .......... ..........
AB971336.1 An. wejchoochotei HCE2(1)          ...T...... .....T.... .......... A......... .......... ..........
AB971337.1 An. wejchoochotei HCE3(1)          ...T...... .....T.... .......... A......... .......... ..........
AB971338.1 An. wejchoochotei HCE4(1)          ...T...... .....T.... .......... A......... .......... ..........
```

```
                                              4444444444 4444444444 4444444444 4444444444 4444444444 4444444444
                                              2222222223 3333333334 4444444445 5555555556 6666666667 7777777778
                                              1234567890 1234567890 1234567890 1234567890 1234567890 1234567890
AB331574.1 An. barbirostris ACB1              TTCAATTTTA GGAGCAGTAA ATTTTATTAC TACTGTTATT AATATACGAT CACCAGGAAT
AB331575.1 An. barbirostris ACB2              .......... .......... .......... .......... .......... ..........
AB331576.1 An. barbirostris ACB3              .......... .......... .......... .......... .......... ..........
AB331577.1 An. barbirostris AUB2              .......... .......... .......... .......... .......... ..........
AB331578.1 An. barbirostris AUB6              .......... .......... .......... .......... .......... ..........
AB331579.1 An. barbirostris AUB10             .......... .......... .......... .......... .......... ..........
AB331580.1 An. barbirostris AUB11             .......... .......... .......... .......... .......... ..........
AB435997.1 An. barbirostris ALpA1             .......... ..G....... .......... .......... ........T. .......T..
AB436002.1 An. barbirostris AUdA8             .......... .......... .......... .......... ........T. .......T..
AB436007.1 An. barbirostris AUbB1             .......... ..G....... .......... .......... ........T. .......T..
AB436014.1 An. barbirostris ARbA3             .......... ..G....... .......... .......... ........T. .......T..
AB436015.1 An. barbirostris ARbB4             .......... ..G....... .......... .......... ........T. .......T..
AB331570.1 An. barbirostris APA13             .......... ..G....... .......... .......... ........T. .......T..
AB331571.1 An. barbirostris APA14             .......... ..G....... .......... .......... ........T. .......T..
AB331572.1 An. barbirostris APA18             .......... ..G....... .......... .......... ........T. .......T..
AB362238.1 An. barbirostris AKA2              .......... .......... .......... .......... ........T. ..........
AB362239.1 An. barbirostris AKA3              .......... .......... .......... .......... ........T. ..........
AB362240.1 An. barbirostris AKA5              .......... .......... .......... .......... ........T. ..........
AB373942.1 An. barbirostris ACA9              .......... .......... .......... .......... ........T. .......T..
AB373943.1 An. barbirostris ACA13             .......... .......... .......... .......... ........T. .......T..
AB373944.1 An. barbirostris ACA18             .......... .......... .......... .......... ........T. .......T..
AB331582.1 An. campestris HCB9                .......... .......... .......... .......... ........T. ..........
AB331583.1 An. campestris HCB10               .......... .......... .......... .......... ........T. ..........
AB331584.1 An. campestris HCB13               .......... .......... .......... .......... ........T. ..........
AB331585.1 An. campestris HCE6                .......... .......... .......... .......... ........T. ..........
AB331586.1 An. campestris HCE7                .......... .......... .......... .......... ........T. ..........
AB331587.1 An. campestris HCE8                .......... .......... .......... .......... ........T. ..........
AB331588.1 An. campestris HCE16               .......... .......... .......... .......... ........T. ..........
AB971336.1 An. wejchoochotei HCE2(1)          .......... .......... .......... .......... ........T. ..........
AB971337.1 An. wejchoochotei HCE3(1)          .......... .......... .......... .......... ........T. ..........
AB971338.1 An. wejchoochotei HCE4(1)          .......... .......... .......... .......... ........T. ..........
```

```
                                          4444444444 4444444445 5555555555 5555555555 5555555555 5555555555
                                          8888888889 9999999990 0000000001 1111111112 2222222223 3333333334
                                          1234567890 1234567890 1234567890 1234567890 1234567890 1234567890
AB331574.1 An. barbirostris ACB1          TACTCTTGAT CGAATACCTT TATTTGTTTG ATCTGTAGTT ATTACAGCAG TTCTTTTATT
AB331575.1 An. barbirostris ACB2          .......... .......... .......... .......... .......... ..........
AB331576.1 An. barbirostris ACB3          .......... .......... .......... .......... .......... ..........
AB331577.1 An. barbirostris AUB2          .......... .......... .......... .......... .......... ..........
AB331578.1 An. barbirostris AUB6          .......... .......... .......... .......... .......... ..........
AB331579.1 An. barbirostris AUB10         .......... .......... .......... .......... .......... ..........
AB331580.1 An. barbirostris AUB11         ...C...... .......... .......... .......... .......... ..........
AB435997.1 An. barbirostris ALpA1         .......... .......... .......... G......... .......... ..........
AB436002.1 An. barbirostris AUdA8         .......... .......... .......... .......... .......... ..........
AB436007.1 An. barbirostris AUbB1         .......... .......... .......... .......... .......... ..........
AB436014.1 An. barbirostris ARbA3         .......... .......... .......... .......... .......... ..........
AB436015.1 An. barbirostris ARbB4         .......... .......... .......... G......... .......... ..........
AB331570.1 An. barbirostris APA13         .......... .......... .......... .......... .......... ..........
AB331571.1 An. barbirostris APA14         .......... .......... .......... .......... .......... ..........
AB331572.1 An. barbirostris APA18         .......... .......... .......... .......... .......... ..........
AB362238.1 An. barbirostris AKA2          ....T.A... ........A. .G.....C.. .......... .........A ..........
AB362239.1 An. barbirostris AKA3          ....T.A... ........A. .G.....C.. .......... .........A ..........
AB362240.1 An. barbirostris AKA5          ....T.A... ........A. .G.....C.. .......... .........A ..........
AB373942.1 An. barbirostris ACA9          .......... .......... .......... .......... .......... ..........
AB373943.1 An. barbirostris ACA13         .......... .......... .......... .......... .......... ..........
AB373944.1 An. barbirostris ACA18         .......... .......... .......... .......... .......... ..........
AB331582.1 An. campestris HCB9            .......... .......... .......... .......... .......... ..........
AB331583.1 An. campestris HCB10           .......... .......... .......... .......... .......... ..........
AB331584.1 An. campestris HCB13           .......... .......... .......... .......... .......... ..........
AB331585.1 An. campestris HCE6            .......... .......... .......... .......... .......... ..........
AB331586.1 An. campestris HCE7            .......... .......... .......... .......... .......... ..........
AB331587.1 An. campestris HCE8            .......... .......... .......... .......... .......... ..........
AB331588.1 An. campestris HCE16           .......... .......... .......... .......... .......... ..........
AB971336.1 An. wejchoochotei HCE2(1)      .......... .......... .......... .......... .......... ..........
AB971337.1 An. wejchoochotei HCE3(1)      .......... .......... .......... .......... .......... ..........
AB971338.1 An. wejchoochotei HCE4(1)      .......... .......... .......... .......... .......... ..........


                                          5555555555 5555555555 5555555555 5555555555 5555555555 5555555556
                                          4444444445 5555555556 6666666667 7777777778 8888888889 9999999990
                                          1234567890 1234567890 1234567890 1234567890 1234567890 1234567890
AB331574.1 An. barbirostris ACB1          ATTATCTTTA CCAGTATTAG CAGGAGCAAT TACTATATTA TTAACTGATC GAAATTTAAA
AB331575.1 An. barbirostris ACB2          .......... .......... .......... .......... .......... ..........
AB331576.1 An. barbirostris ACB3          .......... .......... .......... .......... .......... ..........
AB331577.1 An. barbirostris AUB2          .......... .......... .......... .......... .......... ..........
AB331578.1 An. barbirostris AUB6          .......... .......... .......... .......... .......... ..........
AB331579.1 An. barbirostris AUB10         .......... .......... .......... .......... .......... ..........
AB331580.1 An. barbirostris AUB11         .......... .......... .......... .......... .......... ..........
AB435997.1 An. barbirostris ALpA1         .......... .......... .......... .......... .......... ..........
AB436002.1 An. barbirostris AUdA8         .......... .......... .......... .......... .......... ..........
AB436007.1 An. barbirostris AUbB1         .......... .......... .......... .......... .......... ..........
AB436014.1 An. barbirostris ARbA3         .......... .......... .......... .......... .......... ..........
AB436015.1 An. barbirostris ARbB4         .......... ..G....... .......... .......... .......... ..........
AB331570.1 An. barbirostris APA13         .......... .......... .......... .......... .......... ..........
AB331571.1 An. barbirostris APA14         .......... .......... .......... .......... .......... ..........
AB331572.1 An. barbirostris APA18         .......... .......... .......... .......... .......... ..........
AB362238.1 An. barbirostris AKA2          .......... .......... .......... .......... ........C. ..........
AB362239.1 An. barbirostris AKA3          .......... .......... .......... .......... ........C. ..........
AB362240.1 An. barbirostris AKA5          .......... .......... .......... .......... ........C. ..........
AB373942.1 An. barbirostris ACA9          .......... .......... .T..T..... .......... .......... ..........
AB373943.1 An. barbirostris ACA13         .......... .......... .T..T..... .......... .......... ..........
AB373944.1 An. barbirostris ACA18         .......... .......... .T..T..... .......... .......... ..........
AB331582.1 An. campestris HCB9            .......... .....G.... .......... .......... .......... ..........
AB331583.1 An. campestris HCB10           .......... .....G.... .......... .......... .......... ..........
AB331584.1 An. campestris HCB13           .......... .....G.... .......... .......... .......... ..........
AB331585.1 An. campestris HCE6            .......... .....G.... .......... .......... .......... ..........
AB331586.1 An. campestris HCE7            .......... .....G.... .......... .......... .......... ..........
AB331587.1 An. campestris HCE8            .......... .....G.... .......... .......... .......... ..........
AB331588.1 An. campestris HCE16           .......... .....G.... .......... .......... .......... ..........
AB971336.1 An. wejchoochotei HCE2(1)      .......... .....G.... .......... .......... .......... ..........
AB971337.1 An. wejchoochotei HCE3(1)      .......... .....G.... .......... .......... .......... ..........
AB971338.1 An. wejchoochotei HCE4(1)      .......... .....G.... .......... .......... .......... ..........
```

	6666666666	6666666666	6666666666	6666666666	6666666666	66666666
	0000000001	1111111112	2222222223	3333333334	4444444445	55555555
	1234567890	1234567890	1234567890	1234567890	1234567890	12345678
AB331574.1 *An. barbirostris* ACB1	TACATCATTT	TTTGATCCTG	CAGGAGGAGG	AGATCCAATT	TTATATCAAC	ATTTATTT
AB331575.1 *An. barbirostris* ACB2	....	....	....	....	....	....
AB331576.1 *An. barbirostris* ACB3	....	....	....	....	....	....
AB331577.1 *An. barbirostris* AUB2	....	....	....	....	....	....
AB331578.1 *An. barbirostris* AUB6	....	....	....	....	....	....
AB331579.1 *An. barbirostris* AUB10	....	....	....	....	....	....
AB331580.1 *An. barbirostris* AUB11	....	....	....	....	....	....
AB435997.1 *An. barbirostris* ALpA1	......T..C	....	.......G..	T..C......	....	....
AB436002.1 *An. barbirostris* AUdA8	......T..C	....	....	T..C......	....	....
AB436007.1 *An. barbirostris* AUbB1	......T..C	....	....	T.........	....	....
AB436014.1 *An. barbirostris* ARbA3	......T..C	....	....	T..C......	....	....
AB436015.1 *An. barbirostris* ARbB4	......T..C	....	.......G..	T.........	....	....
AB331570.1 *An. barbirostris* APA13	......T..C	....	....	T..C......	....	....
AB331571.1 *An. barbirostris* APA14	......T..C	....	....	T..C......	....	....
AB331572.1 *An. barbirostris* APA18	......T..C	....	....	T..C......	....	....
AB362238.1 *An. barbirostris* AKA2	...C......	.....C....	....	G.........	....	....
AB362239.1 *An. barbirostris* AKA3	...C......	.....C....	....	G.........	....	....
AB362240.1 *An. barbirostris* AKA5	...C......	.....C....	....	G.........	....	....
AB373942.1 *An. barbirostris* ACA9	......C..C	.....C....	....	...C......	....	....
AB373943.1 *An. barbirostris* ACA13	......C..C	.....C....	....	...C......	....	....
AB373944.1 *An. barbirostris* ACA18	......C..C	.....C....	....	...C......	....	....
AB331582.1 *An. campestris* HCB9	......T..C	.....C....	....	...C......	....	....
AB331583.1 *An. campestris* HCB10	......T..C	.....C....	....	...C......	....	....
AB331584.1 *An. campestris* HCB13	......T..C	.....C....	.T........	...C......	....	....
AB331585.1 *An. campestris* HCE6	......T..C	.....C....	....	...C......	....	....
AB331586.1 *An. campestris* HCE7	......T..C	.....C....	....	....	....	....
AB331587.1 *An. campestris* HCE8	......T..C	.....C....	....	...C......	....	....
AB331588.1 *An. campestris* HCE16	......T..C	.....C....	....	...C......	....	....
AB971336.1 *An. wejchoochotei* HCE2(1)	......T..C	.....C....	....	...C......	....	....
AB971337.1 *An. wejchoochotei* HCE3(1)	......T..C	.....C....	....	....	....	....
AB971338.1 *An. wejchoochotei* HCE4(1)	......T..C	.....C....	....	...C......	....	....

Figure S2. Alignments of the *COI* sequences of the 111 specimens and GenBank sequences included in the study. Dashes (–) indicate alignment gaps and dots (·) indicate identity of nucleotides within the alignment.

	1111 1111111111 1112222222 2222333333 3333333333 3333334444 4444444444 4445555555 5555555666 666666
	111245667 7788990011 2334466778 8990125556 6678000112 2233444455 6677780015 5556667788 8990012344 5578899011 113334
	3469505470 3656143735 6692869587 9675431364 5945124032 5817013958 1434610381 4780390214 7365859817 0341925103 691792
AB971340.1_*An. wejchoochotei* HCE7(1) (Chiang Mai: Thailand)	TGCAATTTTA ATAGTATAAA TTCTTAAACG CATTATTATT TTAGGGTTAT CTTTTGGTAA TTAGATATAA TACAATCTAT ATATTGAAGA AATTATCCAA ACTTTT
AB971339.1_*An. wejchoochotei* HCE5(1) (Chiang Mai: Thailand)	....
AB436124.1_*An. campestris* HSkE2 (Sa Kaeo: Thailand)	....
MT394457_*An. wejchoochotei* SS1 (Chiang Mai: Thailand)	....
MT450816_*An. wejchoochotei* SS2 (Chiang Mai: Thailand)	....C..... ....G ....
MT450817_*An. wejchoochotei* SS3 (Chiang Mai: Thailand)	....
MT450818_*An. wejchoochotei* SS5 (Chiang Mai: Thailand)	....C. ....G. ....
MT450819_*An. wejchoochotei* SS4 (Chiang Mai: Thailand)	....
MT450820_*An. wejchoochotei* SS6 (Chiang Mai: Thailand)	....A...G. ....G... .... .T....
MT450821_*An. wejchoochotei* MO1 (Chiang Mai: Thailand)	....C. ....G. ....
MT450822_*An. wejchoochotei* MO2 (Chiang Mai: Thailand)	....G. ....
MT394456_*An. wejchoochotei* MO3 (Chiang Mai: Thailand)	....C..... ....G ....
MT450823_*An. wejchoochotei* MO4 (Chiang Mai: Thailand)	....C. ....G. ....
MT450824_*An. wejchoochotei* MO5 (Chiang Mai: Thailand)	....
MT450825_*An. wejchoochotei* MO6 (Chiang Mai: Thailand)	....G. ....
MT450826_*An. wejchoochotei* MO7 (Chiang Mai: Thailand)	....G. ....
MT450827_*An. wejchoochotei* Banti1 (Lamphun: Thailand)	....
MT450828_*An. wejchoochotei* Banti2 (Lamphun: Thailand)	....
MT394452_*An. wejchoochotei* Banti3 (Lamphun: Thailand)	....C. ....G. ....
MT450829_*An. wejchoochotei* Banti4 (Lamphun: Thailand)	....G. ....
MT450830_*An. wejchoochotei* Banti5 (Lamphun: Thailand)	....G. ....
MT450831_*An. wejchoochotei* Banti6 (Lamphun: Thailand)	....G. ....
MT450832_*An. wejchoochotei* Banti7 (Lamphun: Thailand)	....A...G. ....G... .... .T....
MT450833_*An. wejchoochotei* Banti8 (Lamphun: Thailand)	....A..G. ....G... ....A. ....C...
MT450834_*An. wejchoochotei* CB305 (Chanthaburi: Thailand)	....G. ....G .... .T....
MT450835_*An. wejchoochotei* CB308 (Chanthaburi: Thailand)	....
MT450836_*An. wejchoochotei* CB270 (Chanthaburi: Thailand)	....
MT394453_*An. wejchoochotei* CB297 (Chanthaburi: Thailand)	....G. ....
AB436105.1_*An. campestris* HCmB18 (Chiang Mai: Thailand)	....A...... ....
MT450813_*An. wejchoochotei* MH1 (Chiang Mai: Thailand)	....C. ....G. ....
MT394455_*An. wejchoochotei* MH2 (Chiang Mai: Thailand)	....G. ....G... G......... .T....
MT450814_*An. wejchoochotei* HD1 (Chiang Mai: Thailand)	....
MT450815_*An. wejchoochotei* HD2 (Chiang Mai: Thailand)	....G. ....G... .... .T....
MT394454_*An. wejchoochotei* HD3 (Chiang Mai: Thailand)	....A...G. ....
MN264217.1_*An. barbirostris* seqbb2 (India)	---------- ---------- ---------T ....G. ....G... G......... .T....
KF564681.1_*An. barbirostris* EHI/10-100510 (Singapore)	---------- ---------- ---------- -......... ....A... ......G... ....T...G. ....AG ....T.. T.C...
AB331571.1_*An. barbirostris* APA14 (Phetchaburi: Thailand)	..A.....C. .A........ ....A ...C...... ....A...G. ......A... ....G. ....T..... ....A. ....T.. T.....
AB971327.1_*An. saeungae* BCLp16(1) (Lampang: Thailand)	..A.....C. .A........ ....CG...A ...C...... .A..A..... ....C.A... ....G. ....T..... ....A. ....T.. TT....
MT394447_*An. saeungae* Camb3-20 (Ratanakiri: Cambodia)	..A.G...C. .A........ .....G...A ...C...... ....A...G. ......A... ....G. ....T..... ....A. ....T.. T.....
MT450756_*An. saeungae* UB116_2 (Ubon Ratchathani: Thailand)	G.A.....C. .A........ ....A ...C...... ....A..... ....C..... ....T..... ....A. ....T.. T.....
MT450781_*An. saeungae* UB2557 (Ubon Ratchathani: Thailand)	..A.....C. .A........ .....G...A ...C...... ....A..... ....C.A... ....G. ....T..... ....A. ....T.. T.....
MT394450_*An. saeungae* UB1001 (Ubon Ratchathani: Thailand)	..A.....C. .A........ .....G...A ...C...... ....A...G. ......A... ....G. ...GT..... ....A. ....T.. T.....
AB331570.1_*An. barbirostris* APA13 (Phetchaburi: Thailand)	..A.....C. .A........ G....G...A ...C.....G C...A...G. ..A.C.A... ....G. ....T..... ....A. ....T.. T.....
AB971326.1_*An. saeungae* BCLp12(1) (Lampang: Thailand)	..A.....C. .A........ .....G...A ...C...... ....A...G. ......A..G ....G. ....T..... ....A. ....T.G T.....
MT394449_*An. saeungae* Tak3 (Tak: Thailand)	..A.....C. .A........ .....GG..A ...C...... ....A..... ....C.A... ....G. ....T..... ....A. ....TTG T.....
MT394448_*An. saeungae* LP1 (Lampang: Thailand)	..A.....C. .A........ .....G...A ...C...... ....A...G. ......A... ....G. ....T..... ..G....GA. ...A...TTG T.....
MT450800_*An. saeungae* LP2 (Lampang: Thailand)	..A.....C. .A........ .....G...A ...C...... ....A...G. ......A... ....G. ....T..... ..G....GA. ...A...TTG T.....
MN166188.1_*An. barbirostris* MS4 (India)	....C... ..T......A ....GC..A. ....A..... T......... A......... .G..T..... ....A. .....C...G ------
MT394440_*An. barbirostris* s.l. GE (Gelephu: Bhutan)	....C... ..T......A ....GC..A. ....A..... T......... A......... .G..T..... ....A. .....C...G TT....
MN254802.1_*An. barbirostris* seqbb1 (India)	---------- ---------- ---------T ....GC..A. ....A..... T......... A......... .G..T..... ....GA. .....C...G TT....
AY729982.1_*An. barbirostris* (Pondicherry: India)	---------- ---------- -----...TT ....GC..A. ....A..... T......... A......... .G..T..... ....GA. .....C...G TT....
AB373942.1_*An. barbirostris* ACA9 (Chiang Mai: Thailand)	.....A.... ..G....... ....TA ......C... ....A.C.G. T......... ...T...... ....T..... ....AT T....C.... ......
AB373944.1_*An. barbirostris* ACA18 (Chiang Mai: Thailand)	.....A.... ..G....... ....TA T.....C... ....A...G. T......... ...T...... ....T..... ....AT T....C.... ......
AB373943.1_*An. barbirostris* ACA13 (Chiang Mai: Thailand)	.....A.... ..G....... ....TA ....A...G. T......... ...T.A.... ....T..... ....AT T....C.... ......
MT394441_*An. barbirostris* s.s. CD1 (Chiang Mai: Thailand)	.....A.... ..G....... ....TA ......C... ....A...G. T......... ....T..... ....AT T....C.... .T....
MT394442_*An. barbirostris* s.s. CD2 (Chiang Mai: Thailand)	.....A.... ..G....... ....TA ......C... ....A...G. T......... ....T..... ....AT T....C.... .T....
MT394430_*An. barbirostris* UNHAS1 (South Sulawesi: Indonesia)	....G....... ....TA ......C... ....A..... T......... ....G ....T..... ....AT T......T.. .TC...
MT450805_*An. barbirostris* UNHAS2 (South Sulawesi: Indonesia)	....G....... ....TA ......C... ....A..... T......... ....G ....T..... ....AT T......T.. .TC...
MT394433_*An. barbirostris* Wulai1 (West Sulawesi: Indonesia)	....G....... ....TA ......C... ....A..... TC........ ....T..... ....T T......T.. .T....
MT394429_*An. barbirostris* Pucak1 (South Sulawesi: Indonesia)	....G....... ....TA ......C.C. .... TC.......G ....T..... ....AT T......T.. .T....
MT450806_*An. barbirostris* Pucak2 (South Sulawesi: Indonesia)	....G....... ....TA ......C.C. ....A..... TC.......G ....T..... ....AT T......T.. .T....
MT394431_*An. barbirostris* Ban1 (South Sulawesi: Indonesia)	....G....... ....TA ......C... .... TC........ ....T..... ....AT T......T.. .T..CA
MT394432_*An. barbirostris* Lak1 (South Sulawesi: Indonesia)	.........G ..G....... ....TA ......C... .... TC........ ....T..... ...A....AT T......T.. .T....
MT450801_*An. barbirostris* Lak2 (South Sulawesi: Indonesia)	....G....... ....TA ......C... .... TC.......G ....T..... ....AT T......T.. .T....
MT450802_*An. barbirostris* Lak3 (South Sulawesi: Indonesia)	....G....... ....TA ......C... .... T......... ....G ....T..... ....AT T......T.. ..C...
MT450803_*An. barbirostris* Lak4 (South Sulawesi: Indonesia)	....G....... ....TA ......C... .... TC........ ....T..... ....AT T......T.. .T....
MT450804_*An. barbirostris* Lak05 (South Sulawesi: Indonesia)	....G....... ....TA ......C.C. ....A..... TC........ ....T...G. ....AT T......T.. .T....

```
MT394451_An. vanderwulpi van7                                          ..T.....T .......... ........T .......C. ....A..... TC....A..G .......... ....T..... ........A. T....A.T.. .TC...
MT450754_An. dissidens UB55 (Ubon Ratchathani: Thailand)               ......A... .......... ..T......A ...C...... ....A..... TAA.C.AA.. C...T..... A......... ........A. .....ATT.. .T....
MT450755_An. dissidens UB116 (Ubon Ratchathani: Thailand)              ......A... .......... ..T......A ...C...... ....A..... TAA.C.AA.. C...T..... A......... ........A. .....ATT.. .T....
MT450757_An. dissidens UB155 (Ubon Ratchathani: Thailand)              ......A... ....C..... ..T......A ...C...... ....A..... TAA.C.AA.. C...T..... A...C..... ........A. .....ATT.. .T....
MT450758_An. dissidens UB567 (Ubon Ratchathani: Thailand)              ......A... .......... ..T......A ...C....C. ....A..... TAA.C.AA.. C...T..... A...C..... ........A. ....CATT.. .T....
MT450759_An. dissidens UB570 (Ubon Ratchathani: Thailand)              ......A... .......... ..T......A ...C....C. ....A..... TAA.C.AA.. C...T..... A......... ........A. .....ATT.. .T....
MT450760_An. dissidens UB751 (Ubon Ratchathani: Thailand)              ......A... .......... ..T......A ...C...... ....A..... TAA.C.AA.. C...T..... A......... .A......A. .....ATT.. ......
MT450761_An. dissidens UB755 (Ubon Ratchathani: Thailand)              ......A... .......... ..T......A ...C....C. ....A..... TAA.C.AA.. C...T..... A......... ........A. .....ATT.. .T....
MT450762_An. dissidens UB873 (Ubon Ratchathani: Thailand)              ......A... .......... ..T......A ...C....C. ....A..... TAA.C.AA.. C...T..... A......... ........A. .....ATT.. .T....
MT450763_An. dissidens UB922 (Ubon Ratchathani: Thailand)              ......A... .......... ..T......A ...C....C. ....A..... TAA.C.AA.. C...T..... A......... ........A. .....ATT.. .T....
MT450764_An. dissidens UB941 (Ubon Ratchathani: Thailand)              ......A... .......... ..T......A ...C....C. ....A..... TAA.C.AA.. C...T..... A......... ........A. .....ATT.. .T....
MT450765_An. dissidens UB986 (Ubon Ratchathani: Thailand)              .T....A... G......... ..T......A ...C....C. ....A..... TAA.C.AC.. C...T..... A......... ........A. .....ATT.. .T....
MT450766_An. dissidens UB1083 (Ubon Ratchathani: Thailand)             ......A... .......... ..T......A ...C...... ....A..... TAAAC.AA.. C...T..C.. A......... ........A. .....ATT.. .T....
MT450767_An. dissidens UB1190 (Ubon Ratchathani: Thailand)             ......A... .......... ..T......A ...C...... ....A..... TAA.C.AA.. C...T..... A......... ........A. .....ATT.. .T....
MT450768_An. dissidens UB1228 (Ubon Ratchathani: Thailand)             ......A... .......... ..T......A ...C.C..C. ....A..... TAA.C.AA.. C...T..... A......... ........A. .....ATT.. .T....
MT450769_An. dissidens UB1363 (Ubon Ratchathani: Thailand)             ......A... .......... .........A ...C...... ....A..... TAA.C.AAG. C...T..... A.T....... ........A. .....ATT.. .T....
MT450770_An. dissidens UB1373 (Ubon Ratchathani: Thailand)             ......A... .......... ..T......A ...C....C. ....A..... TAA.C.AA.. CC..T..... A......... ........A. .....AT... .T....
MT450771_An. dissidens UB1446 (Ubon Ratchathani: Thailand)             ......A... .......... ..T......A ...C....C. ....A..... TAA.C.AA.. C...T..... A......... ........A. .....AT... .T....
MT450772_An. dissidens UB1458 (Ubon Ratchathani: Thailand)             ......A... .......... ..T......A ...C....C. ....A..... TAA.C.AA.. C...T..... A......... ........A. .....ATT.. .T....
MT450773_An. dissidens UB1462 (Ubon Ratchathani: Thailand)             ......A... .......... ..T......A ...C....C. ....A..... TAA.C.AA.. C...T..... A......... ........A. .....ATT.. .T....
MT450774_An. dissidens UB1502 (Ubon Ratchathani: Thailand)             ......A... .......... ..T......A ...C...... ....A..... TAA.CAAA.. C...T..... A......... ........A. .....ATT.. .T....
MT450775_An. dissidens UB1558 (Ubon Ratchathani: Thailand)             ......A... .......... ..T......A ...C....C. ....A..... TAA.C.AA.. C...T..... A......... ........A. .G...ATT.. .T....
MT450776_An. dissidens UB1578 (Ubon Ratchathani: Thailand)             ......A... .......... ..T......A ...C....C. ....A..... TAA.C.AA.. C...T..... A......... ........A. .....ATT.. .T....
MT450777_An. dissidens UB1587 (Ubon Ratchathani: Thailand)             ......A... .......... ..T......A ...C...... ....A..... TAA.C.AA.. C...T..... A......... ........A. .....ATT.. .T....
MT450778_An. dissidens UB1611 (Ubon Ratchathani: Thailand)             ......A... .......... ..T......A ...C....C. ....A..... TAA.C.AA.. C...T..... A......... ........A. .....ATT.. .T....
MT450779_An. dissidens UB2528 (Ubon Ratchathani: Thailand)             ......A... .......... ..T...G..A ...C...... ....A..... TAA.C.AA.G C...T..... A......... ........A. .....ATT.. .T....
MT450780_An. dissidens UB2554 (Ubon Ratchathani: Thailand)             ......A... .......... ..T......A ...C....C. ....A..... TAA.C.AA.. C...T..... A......... ........A. .....ATT.. .T....
MT450782_An. dissidens UB4609 (Ubon Ratchathani: Thailand)             ......A... .......... ..T......A ...C....C. ....A..... TAA.C.AA.. C...T..... A......... ........A. .....ATT.. .T....
MT450783_An. dissidens UB5766 (Ubon Ratchathani: Thailand)             ......A... .......... ..T......A ...C....C. ....A..... TAA.C.AA.. C...T..... A......... ........A. .....ATT.. .T....
MT450784_An. dissidens UB5767 (Ubon Ratchathani: Thailand)             ......A... .......... ..T......A ...C....C. ....A..... TAA.C.AA.. C...T..... A......... ........A. .....ATT.. .T....
MT450785_An. dissidens UB7237 (Ubon Ratchathani: Thailand)             ......A... .......... ..T......A ...C....C. ....A..... TAA.C.AA.. C...T..... A......... ........A. .....ATT.. .T....
MT450786_An. dissidens UB7448 (Ubon Ratchathani: Thailand)             ......A... .......... ..T......A ...C....C. ....A..... TAA.C.AA.. C...T..... A......... ........A. .....ATT.. .T....
MT450787_An. dissidens UB7574 (Ubon Ratchathani: Thailand)             ......A... .......... ..T......A ...C...... ....A..... TAA.C.AC.. C...T..... A......... ........A. .....ATT.. .T....
MT450788_An. dissidens UB8282 (Ubon Ratchathani: Thailand)             ......A... .......... ..T......A ...C.C..C. ....A..... TAA.C.AA.. C...T..... A......... ........A. .....ATT.. .T....
MT450789_An. dissidens UB8747 (Ubon Ratchathani: Thailand)             ......A... .......... ..T......A ...C....C. ....A..... TAA.C.AA.. C...T..... A......... ........A. .G...ATT.. .T....
MT450790_An. dissidens UB10337 (Ubon Ratchathani: Thailand)            ......A... .......... ..T......A ...C....C. ....A..... TAA.C.AA.. C...T..... A......... ........A. .....AT... .T....
MT450791_An. dissidens UB10477 (Ubon Ratchathani: Thailand)            ......A... .......... ..T......A ...C...... ....A..... TAA.C.AA.. C...T..... A......... ........A. .....ATT.. .T....
MT450792_An. dissidens UB10837 (Ubon Ratchathani: Thailand)            ......A... .......... ..T......A ...C...... ....A..... TAA.C.AA.. C...T..... A......... ........A. .....GTT.. .T....
MT394446_An. dissidens UB1171 (Ubon Ratchathani: Thailand)             ....G.A... .......... ......G.TA ...A...... ..G.A..... TAA.C.AA.. C...T....G A......... ........A. .....AT... ......
MH425429.1_An. barbirostris Viet_D-4 (Vietnam)                         ......A... .......... ..T......A ...C...... ....A..... TCC.C.AA.. C...T..... A......... ........A. .....ATT.. .T....
MH425437.1_An. barbirostris Viet_D-12 (Vietnam)                        ......A... .....T.GTG ..T......A ...C...... ....A..... TCC.C.AA.. C...T..... A......... ........A. .....ATT.. .T....
MH425426.1_An. barbirostris Viet_D-1 (Vietnam)                         ......A... .......... ..T...G..A ...C...... ....A..... TAA.C.AA.G C...T..... A......... ........A. .....ATT.. .T....
MT450795_An. dissidens Tak30 (Tak: Thailand)                           ......A... .......... ..T.....TA ...C....C. ....A..... TAA.C.AA.. C...T..... A......... ........A. .....ATT.. .T....
MT394445_An. dissidens Tak32 (Tak: Thailand)                           ......A... ...A...... ..T...G..A ..C....... ....A..... TAA.C.AA.G C...T..... A......... ........A. .....ATT.. .T....
MT450796_An. dissidens Tak34 (Tak: Thailand)                           ......A... .......... .........A ...C...... ....A..... TAA.C.AA.. C...T....G A......... ........A. .....ATT.. .T....
MH425428.1_An. barbirostris Viet_D-3 (Vietnam)                         ......A... .......... ..T......A ...C....A. ....A..... TAA.C.AA.. C...T..... A......... ........A. .....AT... .T....
MH425427.1_An. barbirostris Viet_D-2 (Vietnam)                         ......A... .......... ..T......A ...C....C. ....A..... TAA.C.AA.. C...T..... A......... ........A. .....GTT.. .T....
LC333240.1_An. dissidens DSC13 (Ubon Ratchathani: Thailand)            ......A... .......... ..T......A ...C....C. ....A..... TAA.C.AA.. C...T..... A......... ........A. .....ATT.. .T....
LC333239.1_An. dissidens DSC12 (Ubon Ratchathani: Thailand)            ......A... .......... ..T......A ...C....C. ....A..... TAA.C.AA.. C...T..... A......... ........A. .....ATT.. .T....
AB331574.1_An. barbirostris ACB1 (Chiang Mai: Thailand)                ......A... .......... ..T......A ...C....C. ....A..... TAA.C.AA.. C...T..... A......... ........A. .....ATT.. .T....
AB331575.1_An. barbirostris ACB2 (Chiang Mai: Thailand)                ......A... .......... ..T......A ...C....C. ....A..... TAA.C.AA.. C...T..... A......... ........A. .....ATT.. .T....
MT394443_An. dissidens Camb1-20 (Ratanakiri: Cambodia)                 ......A... .......... ..T......A ...C....C. ....A..... TAA.C.AA.. C...T..... A......... ........A. .....AT... .T....
MT450807_An. dissidens Camb2-20 (Ratanakiri: Cambodia)                 ......A... .......... ..T......A ...C....C. ....A..... TAA.C.AA.. C...T..... A......... ........A. .....ATT.. .T....
MT450808_An. dissidens Camb1-21 (Ratanakiri: Cambodia)                 ......A... .......... ..T......A ...C...... ....A..... TAA.C.AA.. C...T..... A......... ........A. .....ATT.G .T....
MT450809_An. dissidens Camb2-21 (Ratanakiri: Cambodia)                 ......A... .......... ..T......A ...C....C. ....A..... TAA.C.AA.. C...T..... A......... ........A. .....ATT.. .T....
MT450810_An. dissidens Camb3-21 (Ratanakiri: Cambodia)                 ......A... .......... ..T......A ...C...... ....A..... TAAAC.AA.. C...T..... A......... ........A. .....ATT.. .T....
MT394444_An. dissidens MHS1 (Mae Hong Son: Thailand)                   ......A... .......... ..T......A ...C....C. ....A..... TAA.C.AA.. C...T..... A......... ........A. .....ATT.. .T....
MT450811_An. dissidens MHS2 (Mae Hong Son: Thailand)                   ......A... .......... ..T......A ...C....C. ....A..... TAA.C.AA.. C...T..... A......... ........A. .....ATT.. .T....
MT450812_An. dissidens MHS3 (Mae Hong Son: Thailand)                   ......A... .......... ..T......A ...C....C. ....A..... TAA.C.AA.. C...T..... A......... ........A. .....ATT.. .T....
MK184200.1_An. barbirostris SAB10 (Andaman and Nicobar Islands: India) ......AG.. ....C..... ...C....TT .......... .C..A.CAGA TA....A... ....T..... ......TA.A GC...A---- ---------- ------
MK184151.1_An. barbirostris CNB1 (Andaman and Nicobar Islands: India)  ......AG.. ....C..... ...C....TT .......... .C..A.CAGA TA....A... ....T..... ......TA.A GC...A---- ---------- ------
MK184180.1_An. barbirostris GNB10 (Andaman and Nicobar Islands: India) ......AG.. ....C..... ...C....TT .......... .C..A.CAGA TA....A... ....T..... ......TA.A GC...A---- ---------- ------
MK184190.1_An. barbirostris NMA10 (Andaman and Nicobar Islands: India) ......AG.. ....C..... ...C....TT .......... .C..A.CAGA TA....A... ....T..... ......TA.A GC...A---- ---------- ------
MK184158.1_An. barbirostris CNB8 (Andaman and Nicobar Islands: India)  ......A... ....C..... ...C....TT .......... .C..A.CAGA TA....A... ....T..... ......TA.A GC...A---- ---------- ------
MT394434_An. barbirostris A3 Bhutan1 (Singye: Bhutan)                  ..AG..A... .......... ....A...TT .......... ....A.C..A TA....AA.. ....T..... ......TA.A .C...A..A. ....CGTT.. .T....
MT394435_An. barbirostris A3 Bhutan4 (Singye: Bhutan)                  ..AG..A... .......... ....A...TT ........C. ......C..A TA....AA.. ....T..... ......TA.A .C...A..A. ....CGTT.. .T....
MT450793_An. barbirostris A3 Tak21 (Tak: Thailand)                     ......A... .......... ....A...TT .......... ....A.C..A TA....AA.. ....T..... ......TA.A .C...A..A. ....CAT... GT....
MT450794_An. barbirostris A3 Tak28 (Tak: Thailand)                     ......A... .......... ....A...TT .......... ....A.C..A TA....AA.. ....T..... ......TA.A .C..CA..A. ....CAT... GT....
MT394436_An. barbirostris A3 Tak31 (Tak: Thailand)                     ......A... .......... ....A..GTT .......GC. ....A.C..A TA....AA.. ....T..... ......TA.A .C...A..A. ....CAT... GT.C..
MT394437_An. barbirostris A3 Tak33 (Tak: Thailand)                     ......A... .......... ....A...TT .......... ...AA.C..A TA....AA.. ....T..... ......TA.A .C...A..A. ....CAT... .T....
MT450797_An. barbirostris A3 Tak37 (Tak: Thailand)                     ......A... .......... ....A...TT .G........ ....A.C..A TA....AA.. ....T..... ......TA.A .A...A..A. ....CAT... GT....
```

```
MT394438_An. barbirostris A3 Tak38 (Tak: Thailand)                     ......A... .......... ....A...TT ........C. ....A.C..A TA.C..AA.. ....T..... ......TA.A .C...A..A. ....CAT... GT....
MT450798_An. barbirostris A3 Tak41 (Tak: Thailand)                     ......A... .......... ....A...TT .......... ....A.C..A TA....AA.. ....T..... ......TA.A .C..CA..A. ....CAT... GT....
MT394439_An. barbirostris A3 Tak45 (Tak: Thailand)                     ......A... .......... ....A...TT .......... ....A.C..A TA.A..AA.. ....T..... ......TA.A .C...A..A. ....CATT.. .T....
MT450799_An. barbirostris A3 Tak46 (Tak: Thailand)                     ......A... .......... ....A...TT ........C. ....A.C..A TA.C..AA.. ....T..... ......TA.A .C...A..A. ....CAT... GT....
HM773367.1_An. barbirostris VCRCMM_A19386 (Assam: India)               ---------- ---------- ------..TT .......... ....A.C..A TA....AA.. ....T..... ......TA.A .C...A..A. ....CAT... .T....
AB362238.1_An. barbirostris AKA2 (Kanchanaburi: Thailand)              ......A... .......... .C..A...TT .......... ....A.C..A TA....AA.. ..G.T..... ......TA.A GC...A..A. ..C.CAT... GT....
AB362240.1_An. barbirostris AKA5 (Kanchanaburi: Thailand)              ......A... .......... .C..A...TT .......... ....A.C..A TA....AA.. ....T..... ......TA.A GC...A..A. ..C.CAT... GT....
AB362239.1_An. barbirostris AKA3 (Kanchanaburi: Thailand)              ......A... .......... .C..A...TT .......... ....A.C..A TA....AA.. ....T..... ......TA.A GC...A..A. ..C.CAT... GT....
KF564682.1_An. barbirostris EHI/06-100510 (Singapore)                  ---------- ---------- ---------- -.......C. ....A.C..A TA....AA.. ....T..... ......TA.A .C...A..A. ....CAT... GT....
KF564683.1_An. barbirostris EHI/08-100510 (Singapore)                  ---------- ---------- ---------- -.......C. ....A.C..A TA....AA.. ....T..... ......TA.A .C...A..A. ....CAT... GT....
```
